# Separation method for Pu, Am and Sr in large air filter sample sets

**DOI:** 10.1016/j.mex.2020.100910

**Published:** 2020-05-12

**Authors:** Susanna Salminen-Paatero, Jussi Paatero

**Affiliations:** a Department of Chemistry, Radiochemistry, University of Helsinki, P.O. Box 55, FI-00014, Finland; b Finnish Meteorological Institute, P.O. Box 503, FI-00101 Helsinki, Finland

**Keywords:** Extraction chromatography, Sequential separation, Alpha spectrometry, ICP-MS, radionuclides

## Abstract

A sequential separation method for Pu, Am, and Sr was applied for unusually large sample sets of air filters. The sample sets were combined weekly air filters covering sampling time from three months to five years, while in original method, the analyzed air filters had sampling time of only 1-3 days, containing significantly less organic and inorganic matrix and natural radionuclides. The separation method is based on ashing and wet-ashing, followed by column separations with extraction chromatography and anion exchange. Reference materials IAEA-447, IAEA-384, and NIST-SRM-4353A were analyzed with the modified separation method. IAEA-384 was representing best the composition and radionuclide level in the air filter samples.

• Compared to the original method, sample ashing took considerably longer time (one day vs. several days).
• High concentration of natural radionuclides in the large air filter sample sets interfered first the determination of ^241^Am and ^90^Sr, until an anion exchange step was adopted for removal of ^210^Bi and ^210^Po from Am and Sr fractions.
• After modification, the method is suitable for separating artificial radionuclides ^238,239,240^Pu, ^241^Am, and ^90^Sr from large sample sets of air filters.

## Graphical abstract

**Figure:**
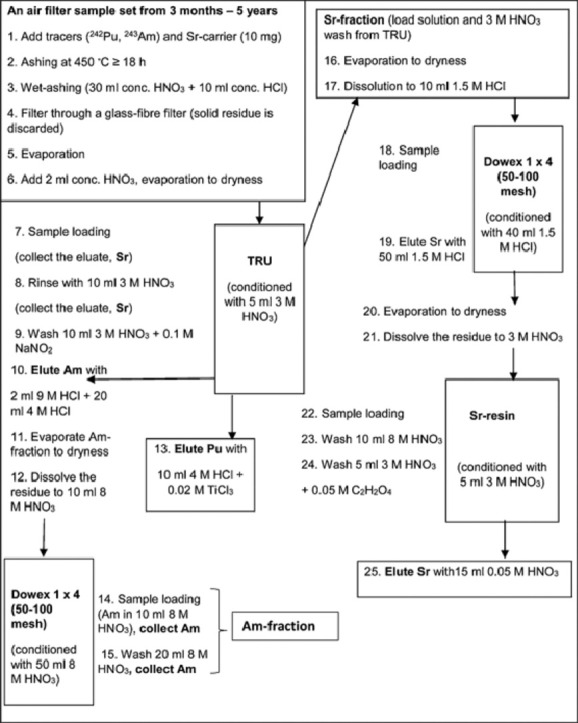

Specifications table

**Table:** 

Subject Area:	Chemistry
More specific subject area:	Environmental radioactivity, radioecology, atmospheric radioactivity
Method name:	Separation of Pu, Am and Sr from large air filter sample sets
Name and reference of original method:	Separation of Pu, Am and Sr from air filters by extraction chromatography. Salminen S. and Paatero J. Concentrations of ^238^Pu, ^239+240^Pu and ^241^Pu in the surface air in Finnish Lapland in 1963. Boreal Environ Res 14:827-836 (2009).
Resource availability:	NA

## Method details

### Radiochemical separation of Pu, Am, and Sr from air filters

The air sampling details from Rovaniemi, Finnish Lapland, as well as the following gamma and total beta measurements have been described in the co-article [1] and in [2]. After gamma measurements, the weekly air filter samples were combined to sets covering sampling period from three months to five years. The selected sampling period depended on the pre-estimation *of* radioactivity level in the air filter samples during the particular year. The air filters were cut into pieces of 2 cm x 2 cm, put into a ceramic evaporation dish covered with a watch glass and ashed in an oven at 450 ˚C for 18 hours. ¼ of the yearly air filter sample set fitted to the evaporation dish at once and 4 evaporation dishes fitted to an oven at once, so it was necessary to continue ashing over 5 days for the largest sample sets containing air filters of five years. For minimizing the sample loss due to transferring the sample from one vessel to another, the remaining filter pieces were added gradually each day to the same evaporation vessels where the ashed residues existed from the particular combined air filter sample set.

The small residue (< 500 mg) was leached with concentrated HCl and HNO_3_ (1:3). This combination dissolves organic and the main part of inorganic matrix in the air filter samples. However, the possible refractory material containing Pu and U isotopes would remain partly undissolved, requiring total dissolution e.g. by lithium metaborate fusion [3], [4], [5], although in some studies also acid leaching has been found to dissolve Pu particles satisfactorily, for example 8 M HNO_3_ with KBrO_3_ [6] or with concentrated HCl [7]. Based on the sampling site and the sampling time period, it was assumed that the investigated air filter samples would contain mostly plutonium from global fallout, i.e. the amount of hot particles in the samples would be minute. Therefore, leaching with concentrated acids would be an adequate treatment for releasing plutonium and americium isotopes into the sample solution.

Before heating the samples, Sr-carrier solution (10 mg/sample) and tracer solutions of ^242^Pu (0.033 Bq/sample) and ^243^Am (0.015 Bq/sample) were added to the samples for determining the radiochemical yield. Based on the annual median Ca concentration at Oulanka, northern Finland in 2010, 0.018 μg/m³, and the reported atmospheric Sr/Ca ratio of 0.0052, it can be calculated that pooled filters of five years contain only 0.2% of the added Sr carrier [8], [9], [10]. A small amount of H_2_O_2_ was added during the last hour of leaching for ensuring the complete oxidation of organic material in the samples. After 6 hours of leaching, the sample solutions were filtered through a glass fibre filter (the tiny residue was discarded) and then evaporated to dryness. After dissolving the residues in 2 ml of conc. HNO_3_ and re-evaporation, the samples were ready for radiochemical separation of ^90^Sr, ^238,239,240^Pu, and ^241^Am.

**Figure 1:**
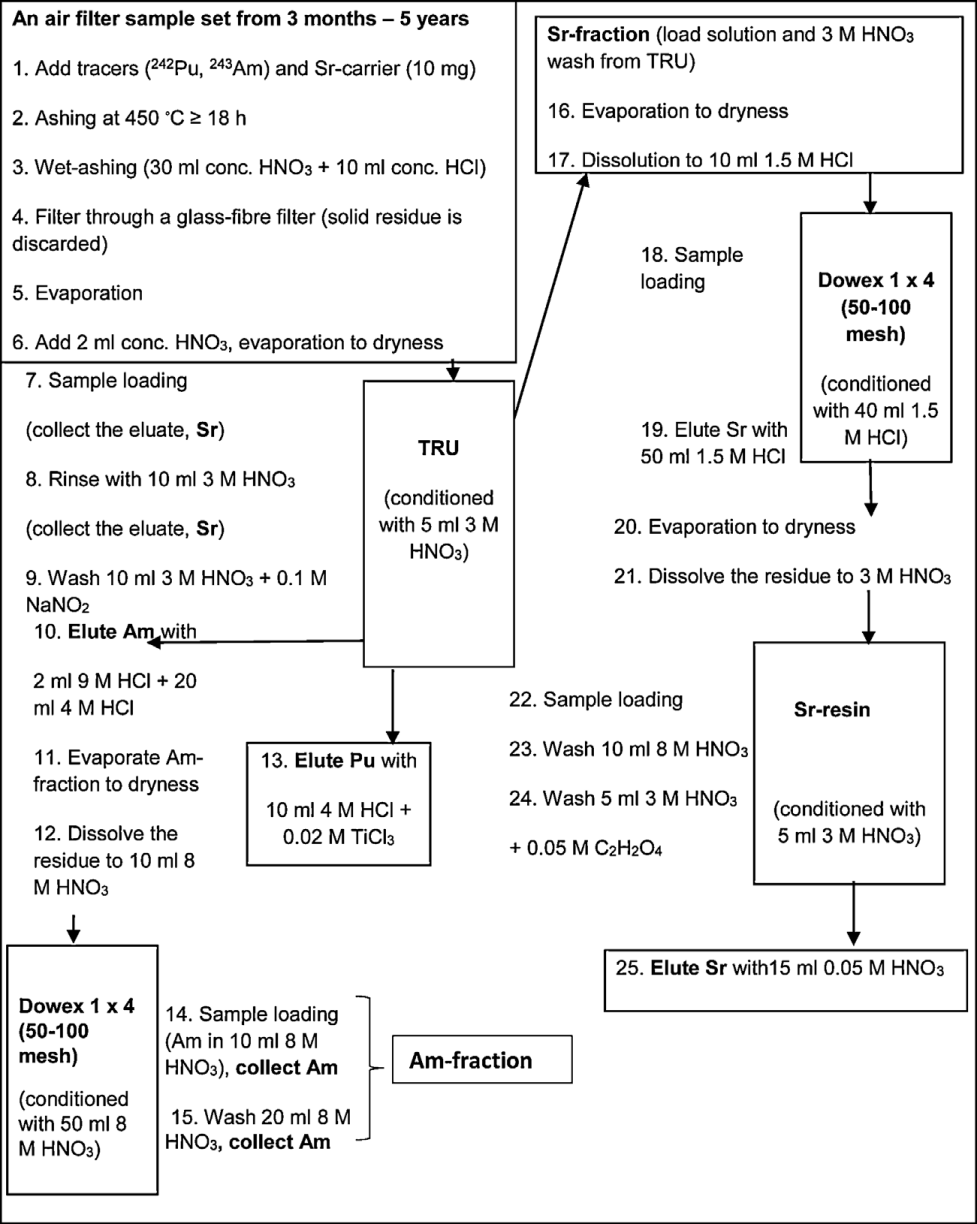
The separation method for determining ^238,239,240^Pu, ^241^Am and ^90^Sr from air filters [2].

The full separation scheme consists of several column separations with anion exchange and extraction chromatography resins ([2] and Figure 1). All of the column separation steps were not originally planned to be included, but were introduced to the scheme later when a need for extra purification of the fractions occurred due to high amount of ^210^Bi and ^210^Po in the air filter samples. The background of extra purification steps will be explained later in the “Modification”-section. The original separation scheme was the same as in Salminen and Paatero [11], containing only extraction chromatography steps for separating Pu, Am and Sr from air filters.

The extraction chromatography columns used were conical 0.8 × 4 cm Econo PolyPrep® columns (BioRad, Hercules, CA, USA), self-packed with 0.7 grams of either TRU® or Sr resin® (TrisKem, Bruz, France) that had been equilibrated with water over night. The elution speed was gravity-controlled. Anion exchange resin Dowex 1 × 4 (Sigma Aldrich, Saint Louis, MO, USA) was loaded to glass Econo® columns (BioRad) of 1 × 15 cm with valves to control elution speed, each column finally containing 8 ml of resin suspension.

^90^Sr, ^238,239,240^Pu and ^241^Am were separated from each other with a TRU® resin column. Then the fraction containing ^90^Sr was further purified with Dowex 1 × 4 and Sr resin® columns. After elution from a Sr resin® column, the samples containing ^90^Sr in 15 ml of 0.05 M HNO_3_ were stored for 3 weeks for allowing the ingrowth of ^90^Y and development of ^90^Y/^90^Sr radioactive equilibrium.

The fraction from the TRU® column containing ^238,239,240^Pu, in 10 ml of 4 M HCl + 0.02 M TiCl_3_, was mixed with Nd-carrier and HF for co-precipitating Pu with NdF_3_. The precipitate was filtered on a membrane filter and glued on a plastic plate as an alpha counting sample.

The fraction from TRU® column containing ^241^Am, in 2 ml 9 M HCl + 20 ml 4 M HCl, was further purified with Dowex 1 × 4 anion exchange column. After anion exchange, evaporation of 8 M HNO_3_ solution containing ^241^Am and re-dissolution of the residue to 1 M HNO_3_, ^241^Am was co-precipitated with NdF_3_ similarly with Pu. The alpha counting sample was produced by filtering the precipitate on a membrane filter.

Four subsamples of three reference materials IAEA-447 (moss-soil, two subsamples of 10 grams and two subsamples of 20 grams), IAEA-384 (Fangataufa sediment, subsamples of 1 gram), and NIST-SRM-4353A (Rocky Flats Soil Number 2, subsamples of 1 gram) were analyzed using the same method as for the air filter samples. The aim in the reference sample analysis was to select the best possible reference material for the large air filter sample sets, analyzed in a unique short-term research project. For this kind of purpose, checking the functionality of the separation method might be enough by analyzing few subsamples of a reference material containing radioactivity level and the composition as similar as possible with the air filter sample sets. A reference material containing adequate concentrations of ^238,239,240^Pu, ^241^Am and ^90^Sr was sought, but there are not many standard reference materials containing all these radionuclides in reasonable activity level and even fewer of them are suitable for air filter analysis regarding the chemical and physical composition. In case of a long-term work, especially in routine analysis of e.g. environmental or nuclear power plant samples, a more comprehensive validation procedure with several reference materials and more subsamples would be required. IAEA-447 was included to testing due to its reasonable activity concentration of ^90^Sr compared to other possible reference materials available. However, as will be discussed later, this reference material was found to be not purpose-fit, since it would require more pre-treatment steps before column separations compared with an air filter, which is a relatively simple sample matrix. The two larger subsamples of IAEA-447 (1 and 2) were split before the column separation for not exceeding the capacity of the extraction chromatography resin columns. Then the purified radionuclide fractions of each subsample halves were reunited after the separation, before preparing alpha counting samples.4 filter and 4 reagent blank samples were analyzed similarly with the reference material and the air filter samples, for observing possible contamination from reagents and glassware, or cross-contamination between the samples.

Ashing was the most time-consuming step in the modified separation method. After ashing, which took 1-5 days depending on the amount of the filters in the particular air filter set, the rest of the separation method for Pu, Am, and Sr took 4-5 working days.

Determining the activity concentrations of ^238,239,240^Pu, ^241^Am, ^90^Sr, and mass ratio ^240^Pu/^239^Pu from the air filter samples

The activity concentrations of ^238,239,240^Pu and ^241^Am in the air filter samples were measured with Alpha Analyst spectrometer (Canberra) with PIPS (Passivated Implanted Planar Silicon) detectors. The counting time was 3-7 days per sample. From the measured activity concentration of ^241^Am, the activity concentration of its beta-emitting mother nuclide ^241^Pu was calculated.

The activity concentration of ^90^Sr in the air filters was determined with a low-background liquid scintillation counter Quantulus 1220 (former Wallac, Perkin Elmer, Turku, Finland) by measuring the activity concentration of its daughter nuclide ^90^Y. Cherenkov counting mode was used and the measuring time was 10 hours per sample. The concentration of stable Sr was measured with ICP-OES for determining the chemical yield of ^90^Sr in a subcontractor's laboratory.

After alpha measurements, the alpha counting samples containing Pu isotopes were dissolved and the samples were further purified with UTEVA and TRU® resins for decreasing ^238^U concentration in the samples and minimizing its tailing effect on ^239^Pu and ^240^Pu in mass spectrometric measurements [12]. The mass ratio ^240^Pu/^239^Pu was determined from the purified Pu fractions by SF-ICP-MS (Sector-Focusing Inductively Couple Plasma-Mass Spectrometry), ELEMENT XR (Thermo Scientific) in ALS Scandinavia Luleå Laboratory. Standard deviation of the mass ratio was calculated from two independent consecutive measurements.

### Results from the analysis of blank and reference material samples

The activity concentrations of ^238^Pu, ^239+240^Pu, ^241^Am, and ^90^Sr determined from three reference materials are presented in Table 1. The activity concentrations of alpha emitters ^238^Pu, ^239+240^Pu, and ^241^Am in analyzed subsamples correspond variably to the reported certified and information values for the respective radionuclides. Uncertainties of experimentally determined activity concentrations are based on one sigma counting error of radioactivity.

**Table 1:** Results from reference sample analysis for ^239+240^Pu, ^238^Pu, ^241^Am, and ^90^Sr. * information value only. # uncertified massic activity by alpha spectrometry. The detection limit D_L_ has been calculated according to the method by Currie [13].

Reference sample and subsample No	Sub-sample mass (g)	A ^241^Am (mBq/g)	Ref. value A ^241^Am (mBq/g) and ref. date	A ^90^Sr (mBq/g)	Ref.value A ^90^Sr (mBq/g) and ref. date
IAEA-447 1	20.0	Unresolvable peaks in the spectrum	* 2.2 ± 0.2, 15^th^ Nov. 2009	7.9 ± 2.8	* 5.0 ± 0.3, 15^th^ Nov. 2009
IAEA-447 2	20.0	“		8.3 ± 2.9	
IAEA-447 3	10.0	“		3.7 ± 1.3	
IAEA-447 4	10.0	“		3.7 ± 1.3	
NIST-SRM-4353A 1	1.0	2.8 ± 0.2	# 2.5 (0.6-5.4), 1^st^ Apr. 1998	< D_L_ 7.1	10.5 ± 1.3 (6.5-15.1), 1^st^ Apr. 1998
NIST-SRM-4353A 2	1.0	3.3 ± 0.2		< D_L_ 7.2	
NIST-SRM-4353A 3	1.0	3.0 ± 0.2		< D_L_ 7.1	
NIST-SRM-4353A 4	1.0	2.6 ± 0.2		< D_L_ 7.1	
IAEA-384 1	1.0	8.3 ± 0.3	7.1 (6.7-7.4), 1^st^ Aug. 1996	< D_L_ 7.2	* 1.7 (1.5-1.9), 1^st^ Aug. 1996
IAEA-384 2	1.0	8.4 ± 0.3		< D_L_ 7.1	
IAEA-384 3	1.0	8.8 ± 0.3		< D_L_ 7.0	
IAEA-384 4	1.0	7.9 ± 0.3		< D_L_ 7.1	

Reference sample and subsample No		A ^239+240^Pu (mBq/g)	Ref. value A ^239+240^Pu (mBq/g) and ref. date	A ^238^Pu (mBq/g)	Ref. value A ^238^Pu (mBq/g) and ref. date
IAEA-447 1	20.0	4.45 ± 0.08	5.30 ± 0.16, 15^th^ Nov. 2009	0.130 ± 0.014	0.150 ± 0.015, 15^th^ Nov. 2009
IAEA-447 2	20.0	4.82 ± 0.12		0.143 ± 0.020	
IAEA-447 3	10.0	< D_L_ 0.060		< D_L_ 0.163	
IAEA-447 4	10.0	4.79 ± 0.08		0.140 ± 0.014	
NIST-SRM-4353A 1	1.0	12.2 ± 0.3	16.8 ± 1.8 (6.0-26.8), 1^st^ Apr. 1998	0.291 ± 0.057	0.278 ± 0.041 (0.18-0.51), 1^st^ Apr. 1998
NIST-SRM-4353A 2	1.0	18.0 ± 0.4		0.518 ± 0.076	
NIST-SRM-4353A 3	1.0	13.8 ± 0.7		0.395 ± 0.114	
NIST-SRM-4353A 4	1.0	12.9 ± 0.4		0.205 ± 0.053	
IAEA-384 1	1.0	106 ± 2	107 (103-110), 1^st^ Aug. 1996	41.8 ± 1.2	39 (38.6-39.6), 1^st^ Aug. 1996
IAEA-384 2	1.0	107 ± 2		39.5 ± 1.4	
IAEA-384 3	1.0	107 ± 2		39.4 ± 1.1	
IAEA-384 4	1.0	103 ± 2		38.2 ± 1.3	

The results were not obtained for ^241^Am in IAEA-447 due to overlapping alpha peaks from ^241^Am and ^243^Am in the spectra, indicating need for further sample purification from lanthanides. This problem concerned only IAEA-447 subsamples, not the air filter samples, due to the following reasons. The subsample masses of IAEA-447 were obviously too high for the separation capacity of the selected method, at least without any pre-purification method (e.g. co-precipitation) before any column separations. The moss-soil reference material contains high amount of complex organic matrix, iron and lanthanides, which all interfere with the radiochemical separation of trivalent ^241^Am^3+^, and, furthermore, it doesn't represent well the chemical composition of air filter residues and aerosol particles. The determined activity concentrations of ^238^Pu (0.130±0.014 - 0.143±0.020 mBq/g) and ^239+240^Pu (4.45±0.08 - 4.82±0.12 mBq/g) were a bit lower than the reference values for IAEA-447, 0.150±0.015 mBq/g and 5.30±0.16 mBq/g, respectively (reference date 15th Nov. 2009). The differences between the experimental and previously reported activity concentration values are small, but they might indicate partly undissolved Pu in the subsamples. This might be due to high sample mass and/or presence of insoluble Pu particles in IAEA-447, both explanations originating from incomplete digestion procedure. This observation further confirms the exclusion of IAEA-447 from using it as a reference material for checking a separation method designed for the air filters.

There was a high variation in the activity concentrations of ^238^Pu (0.205±0.053 – 0.518±0.076 mBq/g) and ^239+240^Pu (12.2±0.3 - 18.0±0.4 mBq/g) among the subsamples of NIST-SRM-4353A. However, the material has published reference ranges for ^238^Pu, 0.18-0.51 mBq/g, and for ^239+240^Pu 6.0-26.8 mBq/g (reference date 1st Apr. 1998). Therefore, widely varying activity concentrations of Pu isotopes are still between the reported ranges, while the exact reference values (0.278±0.041 mBq/g for ^238^Pu and 16.8±1.8 mBq/g for ^239+240^Pu) are inside the range of the determined activity concentrations in this study. The reported activity concentration of ^241^Am in NIST-SRM-4353A has also a wide variation, 0.6-5.4 mBq/g, our values for ^241^Am being at the same level, 2.6±0.2 - 3.3±0.2 mBq/g.

The obtained activity concentration values for ^238^Pu (38.2±1.3 - 41.8±1.2 mBq/g) and ^239+240^Pu (103±2 - 107± 2 mBq/g) match fairly well with the reference values of IAEA-384 reference material, 39 (38.6-39.6) mBq/g and 107 (103-110) mBq/g, respectively (reference date 1st Aug. 1996). The activity concentration values for ^241^Am from the four subsamples of IAEA-384 (7.9±0.3 - 8.8±0.3 mBq/g) were slightly higher than the reference value 7.1 (6.7-7.4) mBq/g. The reference material IAEA-384 contains both ^241^Am and ^241^Pu, a mother nuclide of ^241^Am. It has to be noted, that and the activity concentration of ^241^Pu has a high variation, 41-69 mBq, the mean value being 56±5 mBq/g (again the reference date 1st Aug. 1996) [14]. Consequently, there might be some additional fluctuation in the ingrown ^241^Am activity concentration in the reference material. Furthermore, then the mean value for the activity concentration of ^241^Am (present in the material before the reference date and ingrown from ^241^Pu after the reference date, taking into account the decay of ^241^Am since the reference date) is not as representative measure as the concentration range. Based on this limited data, the sample dissolution method using HNO_3_: HCl dissolved quantitatively Pu and Am isotopes from the sediment matrix, which is also expected for the air filter samples. However, analysis of larger subsample group would enable more statistical performance analysis of the separation method.

The radiochemical recovery of Pu was 14-83% for the eight blank samples and 18-100% for the reference samples. For ^241^Am, the recovery was 61-81% for the eight blanks and 61-82% for the reference samples. Only few counts were seen in the alpha spectra of the blanks excluding the tracer isotope peaks, proving that no cross-contamination occurred during the analytical procedure.

From the reference material subsamples, the activity ratio ^238^Pu/^239+240^Pu and mass ratio ^240^Pu/^239^Pu were also determined (Table 2). The measured activity ratio ^238^Pu/^239+240^Pu agreed quite well with the reference values for IAEA-384 and IAEA-447 but for SRM-4353A, the measured activity ratio ^238^Pu/^239+240^Pu (0.016±0.004 - 0.029±0.008) was higher than the reference value (0.017±0.001). As was discussed with the activity concentration values of Pu isotopes, there is a high variation in the reported activity concentrations of ^238^Pu and ^239+240^Pu in this reference material, being 3-fold for ^238^Pu and 4-fold for ^239+240^Pu. Therefore, it is probable that a wide range of ^238^Pu/^239+240^Pu activity ratio values results from this internal variation in the reference material, in addition to the single reference value. Isotopic fractionation has been observed by using different acids for the sample digestion [15], and it would have been interesting to test whether the use of e.g. total dissolution of the sample by alkali fusion would have produced different Pu activity ratios than obtained by leaching with HNO_3_: HCl. On the other hand, the mass ratios ^240^Pu/^239^Pu in the analyzed subsamples of SRM-4353A and IAEA-384 corresponded well with the reference value. Compared to the alpha spectrometric determination of the ^238^Pu/^239+240^Pu activity ratio the mass spectrometric determination of ^240^Pu/^239^Pu mass ratio may be a more reliable method to test reference materials. However, no further conclusions can be drawn from such a limited dataset.

**Table 2:** Activity ratio ^238^Pu/^239+240^Pu and mass ratio ^240^Pu/^239^Pu in the reference material subsamples. * uncertified value. # [14].

Reference sample and subsample No	Sub-sample mass (g)	A ^238^Pu/ A ^239+240^Pu	Ref. value A ^238^Pu/ A ^239+240^Pu and ref. date	Mass ratio ^240^Pu/^239^Pu	Ref.value ^240^Pu/^239^Pu and ref. date
IAEA-447 1	20.0	0.029 ± 0.003	0.028, 15^th^ Nov. 2009	0.187 ± 0.003	-
IAEA-447 2	20.0	0.030 ± 0.004		0.186 ± 0.009	
IAEA-447 3	10.0	< D_L_		Not determined	
IAEA-447 4	10.0	0.029 ± 0.003		Not determined	
NIST-SRM-4353A 1	1.0	0.024 ± 0.005	0.017 ± 0.001, 1^st^ Apr. 1998	Not determined	0.056 (0.053-0.060), * 1^st^ Apr. 1998
NIST-SRM-4353A 2	1.0	0.029 ± 0.004		Not determined	
NIST-SRM-4353A 3	1.0	0.029 ± 0.008		0.062 ± 0.011	
NIST-SRM-4353A 4	1.0	0.016 ± 0.004		0.063 ± 0.002	
IAEA-384 1	1.0	0.396 ± 0.016	0.364, 1^st^ Aug. 1996	0.049 ± 0.002	0.049 ± 0.001 #
IAEA-384 2	1.0	0.368 ± 0.017		0.051 ± 0.003	
IAEA-384 3	1.0	0.367 ± 0.014		Not determined	
IAEA-384 4	1.0	0.370 ± 0.017		Not determined	

For ^90^Sr, there was high variation among the concentrations of four subsamples IAEA-447 analyzed, from 3.7±1.3 mBq/g to 8.3±2.9 mBq/g, when the information value for IAEA-447 is 5.0±0.3 mBq/g (reference date 15th Nov. 2009) (Table 1). The uncertainty of ^90^Sr activity concentrations is high, due to low activity concentration of ^90^Sr in IAEA-447 and 20% uncertainty in recovery determination of Sr by ICP-OES (uncertainty reported by the subcontractor that was used for determination of stable Sr), and low activity concentration of ^90^Sr in the reference material. Furthermore, the sample matrix of moss-soil IAEA-447 contains iron and organic compounds probably increasing variability of obtained ^90^Sr activity concentration, due to matrix-induced interferences in extraction chromatography. It was observed also with ^90^Sr that IAEA-447 was not the best possible reference material for testing the separation method used for air filters due to the low activity concentration of ^90^Sr in the reference material and complex matrix of moss-soil compared to air filter. Unfortunately, at that moment it was the only available option containing detectable amount of ^90^Sr for this work, in addition to transuranic elements. There was not enough SRM-4353A stock left for this purpose, otherwise this soil might have been easier sample matrix and more representative reference material for air filter analysis.

The chemical recovery of Sr was 45-85% for the blank samples and 68-92% for the reference samples. The activity concentration of ^90^Sr in the eight blank samples were at the background activity level, showing no contamination due to laboratory glassware or reagents used, or from cross-contamination between the samples.

As a summary about the reference sample analyses, the high amount of reference material IAEA-447 required for detecting ^90^Sr caused problems throughout the radioanalytical separation procedure and taking into account the differences in chemical composition between the moss-soil and air filter, this reference material was not suitable for our purposes. NIST-SRM- 4353A sediment was otherwise a potential reference material for the air filter study, but due to the limited stock available, its activity concentration of ^90^Sr was too low to be detected with LSC. Furthermore, the activity concentrations of Pu isotopes can have wide variation in NIST-SRM-4353A. The most representing reference material for the large air filter sample sets was IAEA-384 because it contains relatively easily dissolvable sample matrix, and it has adequate activity concentration of ^241^Am and ^238,239,240^Pu allowing their detection from a small subsample. However, this reference material has very low activity concentration of ^90^Sr and the determination of ^90^Sr can be achieved only by using a large subsample mass, which is not a desirable action in analyzing air filter sample sets with low amount of matrix after ashing the ashless air filters.

For expanding the use of the separation procedure from unique short-term academic research project to a more permanent routine analyses of large number of samples, the validation process should be continued. More subsamples, one or few new purpose-fit reference material and perhaps a total digestion for at least part of the subsamples is required.

### Modification of radioanalytical method due to interference from natural radionuclides

The first attempt was to separate ^90^Sr with TRU® and Sr resin® columns only, but the measured LSC (liquid scintillation counting) spectra of the separated Sr fractions revealed the presence of radioactive impurity and decreasing count rate value during repeat measurements. The sample count rate was decreased to 32-57% of the original value in ∼70 days, this test was made with four samples. The impurity in the Sr fraction was most likely ^210^Bi (t½ 5.01 d) that decays to ^210^Po (t½ 138.4 d), both natural isotopes having concentrations orders of magnitude higher than those of ^90^Sr in the atmosphere [16]. ^210^Bi emits strong beta particles of 1162.1 keV that can be detected with Cherenkov counting, but its daughter ^210^Po is undetectable by Cherenkov counting [17].

^210^Bi and ^210^Po have been reported to interfere ^90^Sr determination with Sr resin® also by Saxén [18]. According to Dietz et al. [19], Pb retains in Sr resin® even more strongly than Sr, and this was also confirmed in the experiments by Saxén [18]. Therefore, Sr resin® not being effective enough in removal of natural radionuclides from Sr fraction as such, it was decided to include an anion exchange column separation to the separation procedure of ^90^Sr, for removing ^210^Bi and ^210^Po from the Sr fraction before Sr resin® separation step. The anion exchange step was modified from the method published by Wallova et al. [20]. After including the anion exchange step into the separation scheme, the count rates of the ^90^Sr peak maintained stable during several months in control measurements of the LSC samples.

The excess amount of ^210^Po in the air filters was also noticed in alpha spectra of ^241^Am after the very first separations, when only TRU® resin was used for the separation of ^241^Am. The radiochemical recovery of ^241^Am was far over 100%. It was decided to use an anion exchange step also for purifying ^241^Am from ^210^Po. Am is not retained from 8 M HNO_3_ to Dowex 1 × 4 resin and it passes immediately through the resin column, while elution of Po is delayed in this acid media, starting only after loading volume of 100 ml, as was proved by Talvitie [21]. After anion exchange step, there was no ^210^Po present in alpha spectra of Am fractions.

It can be concluded that the modifications for the original separation method that was based on the extraction chromatography only, i.e. later added two anion exchange steps, helped with removing excess amount of ^210^Bi and ^210^Po present in the air filter samples before radioactivity determinations of ^90^Sr and ^241^Am. The combined air filter samples containing aerosols from three months to five years had unexpectedly high amount of natural radionuclides. Alternative solutions for the afterwards added anion exchange steps exist, for example, a rinse of 8 M HNO_3_ could have been added to TRU® column for removing Po from the column before eluting Am [22]. Also, the column separation steps could have been in reverse order, i.e. first anion exchange and then extraction chromatography due to better tolerance of impurities of the former. One further option would have been the use of co-precipitation as a pre-concentration method for Sr and actinides before any column separation.
